# Effect of pH and temperature on microbial community structure and carboxylic acid yield during the acidogenic digestion of duckweed

**DOI:** 10.1186/s13068-018-1278-6

**Published:** 2018-10-08

**Authors:** Ozgul Calicioglu, Michael J. Shreve, Tom L. Richard, Rachel A. Brennan

**Affiliations:** 1 Department of Civil and Environmental Engineering, The Pennsylvania State University, 212 Sackett Building, University Park, 16802 USA; 2 Department of Agricultural and Biological Engineering, The Pennsylvania State University, 132 Land and Water Research Building, University Park, PA 16802 USA

**Keywords:** Volatile fatty acids, Acidogenic digestion, Carboxylate platform, Biohydrogen, Duckweed, *Lemna obscura*, Marker-gene survey, Microbial community analysis

## Abstract

**Background:**

Duckweeds (Lemnaceae) are efficient aquatic plants for wastewater treatment due to their high nutrient-uptake capabilities and resilience to severe environmental conditions. Combined with their rapid growth rates, high starch, and low lignin contents, duckweeds have also gained popularity as a biofuel feedstock for thermochemical conversion and alcohol fermentation. However, studies on the acidogenic anaerobic digestion of duckweed into carboxylic acids, another group of chemicals which are precursors of higher-value chemicals and biofuels, are lacking. In this study, a series of laboratory batch experiments were performed to determine the favorable operating conditions (i.e., temperature and pH) to maximize carboxylic acid production from wastewater-derived duckweed during acidogenic digestion. Batch reactors with 25 g/l solid loading were operated anaerobically for 21 days under mesophilic (35 °C) or thermophilic (55 °C) conditions at an acidic (5.3) or basic (9.2) pH. At the conclusion of the experiment, the dominant microbial communities under various operating conditions were assessed using high-throughput sequencing.

**Results:**

The highest duckweed–carboxylic acid conversion of 388 ± 28 mg acetic acid equivalent per gram volatile solids was observed under mesophilic and basic conditions, with an average production rate of 0.59 g/l/day. This result is comparable to those reported for acidogenic digestion of other organics such as food waste. The superior performance observed under these conditions was attributed to both chemical treatment and microbial bioconversion. Hydrogen recovery was only observed under acidic thermophilic conditions, as 23.5 ± 0.5 ml/g of duckweed volatile solids added. More than temperature, pH controlled the overall structure of the microbial communities. For instance, differentially abundant enrichments of *Veillonellaceae acidaminococcus* were observed in acidic samples, whereas enrichments of *Clostridiaceae alkaliphilus* were found in the basic samples. Acidic mesophilic conditions were found to enrich acetoclastic methanogenic populations over processing times longer than 10 days.

**Conclusions:**

Operating conditions have a significant effect on the yield and composition of the end products resulting from acidogenic digestion of duckweed. Wastewater-derived duckweed is a technically feasible alternative feedstock for the production of advanced biofuel precursors; however, techno-economic analysis is needed to determine integrated full-scale system feasibility and economic viability.

**Electronic supplementary material:**

The online version of this article (10.1186/s13068-018-1278-6) contains supplementary material, which is available to authorized users.

## Background

Throughout the industrial era, population growth and increased consumption have resulted in a steady increase in the demand for energy. This demand has been met mainly by nonrenewable fossil-based resources (i.e., coal, crude oil, crude gas) [1], which generate excessive carbon dioxide (CO_2_) emissions and other environmental concerns [2]. As a renewable and sustainable alternative, advanced biomass energy approaches have been attracting increasing attention [3]. However, feedstock sustainability, availability, and affordability issues remain a serious concern. In this context, an environmentally friendly, socially acceptable, and economically feasible biomass crop could overcome the challenges faced by the majority of biofuels on the energy market.

Lemnaceae (duckweeds) represent a family of simple, fast-growing, floating aquatic plants, with five genera (*Landoltia*, *Lemna*, *Spirodela*, *Wolffia*, and *Wolfiella*) and 38 species classified to date [4, 5]. Production of duckweed rich in starch and cellulose can be integrated into wastewater treatment systems, which can improve the economics of the feedstock production process [6]. Moreover, the low lignin content of duckweeds relative to lignocellulosic agricultural residues and traditional energy crops make them an attractive alternative for conversion into bioethanol, since they do not require intensive pretreatment prior to saccharification. Previous studies with duckweed have investigated its use as a feedstock to produce either sugar or syngas intermediates; these two platforms have dominated most of the public funding as well as private investment in advanced biorefineries. Thermochemical conversion of duckweed into syngas demonstrated pathways to gasoline, diesel, and jet fuel [7]. Biochemical conversion of duckweed starch and cellulose into simple sugars and fermentation into alcohols has also been demonstrated, and been applied at both laboratory and pilot scales [8, 9].

A third biomass–biofuel conversion strategy has been termed the carboxylate platform [10]. This platform utilizes mixed cultures for anaerobic degradation of organic matter into carboxylic acid intermediates, a process that has been termed acidogenic digestion. During acidogenic digestion, 2–5 carbon volatile fatty acids (VFAs) are initially produced, and can be converted into longer chain fatty acids consisting of six or more carbon atoms through chain elongation via mixed cultures [11]. These longer chain fatty acids have a higher energy density than short-term VFAs, and are precursors of higher-value chemicals and biofuels such as esters, alcohols, and alkanes [12].

Acidogenic digestion is advantageous over alcohol fermentation due to: (1) the potential to directly utilize feedstocks such as duckweed without requiring pretreatment; (2) production of a single class of end products; (3) the absence of sterilization requirements; and (4) convertibility of longer chain products (3-carbon and higher) into higher-value chemicals and fuels [13]. However, there do not appear to be any prior published studies on processing duckweed through the carboxylate platform.

Carboxylate platform systems also have some drawbacks, such as requiring process control to avoid a shift into methanogenic activity [14]. Methanogenic activity is normally inhibited by either chemical addition or avoiding the conditions which favor methanogens (e.g., maintaining pH outside the range of 5.5–8.5, which methanogens prefer). Indeed, the literature suggests that higher VFA concentrations can be achieved under alkaline conditions of pH 9–pH 10 [15], which should simultaneously suppress methanogenic activity. Under high ammonia concentrations present in reactors at elevated pH, anaerobic bacteria are expected to outcompete methanogenic archaea [16]. However, the behavior of acidogenic microbial consortia at high pH is not well understood.

The objectives of this work were: (1) to evaluate the effect of operating conditions such as temperature and pH on the acidogenic digestion of duckweed; (2) to quantify conversion rates and the associated carboxylic acid yields; and (3) to characterize the dominant microbial taxa present under various operating conditions. This study is the first to determine the performance of duckweed during acidogenic digestion under various operating conditions, with an emphasis on investigating the resulting acidogenic microbial consortia.

## Methods

### Analytical methods

The moisture, total solids (TS), and volatile solids (VS) contents of duckweed and the inocula were determined according to the National Renewable Energy Laboratory (NREL) Laboratory Analytical Procedure (LAP) for biomass and total dissolved solids of liquid process samples [17]. Ash content was measured according to NREL LAP for determination of ash in biomass [18]. Carboxylic acids (i.e., VFAs) were quantified using Gas Chromatography (GC) (SHIMADZU, GC-2010 Plus, Japan) with a flame ionization detector. The final total VFA yields were calculated in terms of acetic acid equivalents per gram duckweed volatile solids added (HAc_eq_ g VS_added_^−1^) [19]. Carbon quantification of samples was performed using a total carbon (TC) analyzer (SHIMADZU, TOC-V CSN, Kyoto, Japan) equipped with solid sample module (SHIMADZU, 5000A, Kyoto, Japan). Total ammonifiable nitrogen (TAN) concentrations were measured by selective electrode method as described in Standard Methods No. 4500 [20], using an ammonia probe (Orion, 9512, USA). Headspace pressure in the reactors was measured using a pressure gauge (Grainger, DPGA-05, USA). If the pressure was found to be negative or zero, no volume readings were performed to avoid disturbance of the headspace gas composition. The gas volumes of reactors were measured using a water displacement device filled with 0.02 M hydrochloric acid. Since the measurement process was quick, the headspace temperature was assumed to be constant and equal to 35 °C [21, 22]. Volume readings were reported at standard temperature and pressure. Volumetric methane (CH_4_) and hydrogen (H_2_) concentrations were determined by extracting headspace from the reactors using a 250 μl gastight syringe (Hamilton, Reno, NV, USA) and injecting onto a GC (SRI Instruments, SRI310C, Torrance, CA, USA) equipped with 6-foot molecular sieve column (SRI 8600-PK2B, USA) in continuous mode at 80 °C with argon as the carrier gas. Volumetric CO_2_ concentrations were quantified using an identical GC equipped with 3-foot silica gel packed column (SRI, 8600-PK1A, USA) in continuous mode at 60 °C with helium as the carrier gas.

### Plant material and growth conditions

Duckweed was collected on May 29, 2016, from an open pond within the effluent spray fields of the Pennsylvania State University Wastewater Treatment Plant (PSU WWTP), a.k.a. the “Living-Filter”, receiving on average (*n* = 9): 2.3 ± 0.5 mg l^−1^ carbonaceous biological oxygen demand; 1.5 ± 0.1 mg l^−1^ phosphorus; 0.6 ± 0.9 mg l^−1^ TAN; 5.8 ± 1.5 mg l^−1^ nitrate; 0.3 ± 0.2 mg l^−1^ nitrite; and 1.3 ± 0.4 mg l^−1^ total Kjeldahl nitrogen. The duckweed species in the pond was identified as a monoculture of *Lemna obscura* (100% sequence identity to accession number GU454331.1, in the NCBI database) through DNA extraction and sequencing as described previously [23]. Prior to using in these experiments, the duckweed was rinsed with tap water and dried at 45 ± 3 °C to a constant weight over 2 days. Duckweed was then analyzed for its moisture (5.0 ± 0.4%), and VS (85.6 ± 0.4%) contents. The composition of duckweed was determined as (% per dry weight): cellulose (11.8 ± 0.9); hemicellulose (20.5 ± 1.0); starch (9.8 ± 0.9); lignin (1.6 ± 1.2); water soluble carbohydrates (19.9 ± 0.2); and crude protein (18.2 ± 0.2) (Dairy One Wet Chemistry Laboratory, Ithaca, NY). A separate batch of duckweed was used to enrich the inoculum, which was previously collected from the same pond, and dried at 45 ± 3 °C to a constant weight. Subsamples of dried duckweed were collected and stored at − 80 °C for future DNA analysis.

### Inoculum

A combination of mesophilic and thermophilic seeds were collected to prepare the inoculum: silage, rumen fluid, and anaerobic wastewater sludge were used as mesophilic seeds; and compost was used as a thermophilic seed [24–26]. Silage and rumen fluid were obtained from the PSU Dairy Farm (University Park, PA). Anaerobic wastewater sludge was obtained from the PSU WWTP’s secondary digester. Compost was obtained from the PSU composting facility. Silage (360 g) and compost (180 g) were each blended separately in 1 l of 25 mM phosphate-buffered saline (PBS) at pH 6.8. Rumen fluid was centrifuged at 2880 rgf for 30 min and the pellet was re-suspended in 1 l of 25 mM PBS at pH 6.8. All three sources were incubated separately overnight at 35 °C. Solids from 1.5 l anaerobic sludge were collected by centrifuging at 2880 rgf for 30 min (Eppendorf, 5804 R, Germany) and were re-suspended in 25 mM PBS at pH 5.0, incubated at 35 °C overnight, and boiled for 1 h to inhibit methanogenic activity [27, 28].

All four sources were screened through a sieve with 150 µm opening. The permeates were blended in equal parts (on a VS basis), previously harvested duckweed was added at a substrate–inoculum ratio of 0.1, and the cultures were acclimated to acidic (pH = 5.3) or basic (pH = 9.2) conditions for 5 and 7 days, respectively, at 35 °C until substantial biogas production was observed. The final slurries were both centrifuged for 30 min at 2880 rgf and the inoculum solids collected. An aliquot of each inoculum was collected and stored at − 80 °C for later DNA extraction. The final compositions of the two inocula were: 84.0 ± 0.1% moisture, and 74.4 ± 1.2% VS of TS for the acidic inoculum; and 84.5 ± 0.2% moisture, and 60.3 ± 0.2% VS of TS for the basic inoculum.

### Acidogenic digestion

Batch reactors (300 ml working volume) were fed with duckweed to achieve a total solids content of 25 g l^−1^, and inoculum was added at an inoculum substrate ratio of 0.1 on a VS basis. Initial pH values were adjusted to either pH 5.3 or pH 9.2. Reactors to be operated under basic conditions were supplemented with 4.0 g l^−1^ sodium carbonate as buffer, which is equivalent to about 5% of the duckweed carbon added and was quantified in the carbon balance accordingly. All reactors were purged with nitrogen gas for 3 min and sealed to provide anaerobic conditions. Reactors were operated under mesophilic (35 °C) or thermophilic (55 °C) conditions for 21 days. Once every 2 days, headspace gas volume and composition were measured, liquid samples were taken, and the pH was adjusted to either 5.3 or 9.2. Test reactors were run in triplicate, and controls (with no substrate) were run in duplicate. The observed biogas values in control reactors were subtracted from those observed in active reactors. The VFA production values, however, were found to be negligible compared to those achieved in active reactors; therefore, they were not subtracted. Duplicate blank reactors (with no inoculum) were also operated to evaluate the acidogenic digestion potential of microorganisms naturally associated with the duckweed, which as previously described was air dried at 45 °C, and not sterilized.

At the end of reactor operation period, samples for microbial community analysis were obtained under axenic conditions. Prior to sacrificing the reactors, 6 ml of liquid was withdrawn and centrifuged sequentially (2 ml at a time) in 2 ml Eppendorf tubes, discarding the supernatant after each cycle to concentrate suspended solids for DNA extraction. Samples for DNA extraction were stored at − 80 °C until processed. The rest of the reactor constituents were wet sieved by pressing through a 340-µm opening. The screenings were analyzed as reactor liquids, and the retentates were analyzed as reactor solids. TAN of the liquids was measured.

### Carbon balance

Initial and final TC concentrations of the headspace, liquids, and solids were reported. Headspace TC was calculated as the sum of CO_2_ and CH_4_ recovered over the 21-day operation period, and the amounts remaining in the headspace at the end of operation. The VFA losses during solids drying were estimated as 95% for acidic reactors and 55% for basic reactors [29]. The sampling losses were calculated as 24 sampling events of 2 ml each. The mass closure has been calculated as the ratio of the final to initial total carbon values (Additional file 1: Table S3).

### DNA extraction, PCR amplification, and high-throughput sequencing

DNA was extracted from approximately 100 mg each of acclimated inoculum and suspended biomass from final (day 21) reactor contents using a Mo Bio PowerSoil DNA extraction kit (MO BIO Laboratories, Inc., Carlsbad, CA, USA) according to the manufacturer’s protocol. Microbial DNA was isolated from dried duckweed samples using the same kit, by adding approximately 25 mg of plant tissue and following the manufacturer’s protocol. The V4 region of the 16S rRNA gene (bacteria and archaea) was PCR-amplified using the primers 515F-Y (5′- GTGYCAGCMGCCGCGGTAA-3′) and 806RB (5′- GGACTACNVGGGTWTCTAAT-3′) [30, 31]. Forward and reverse overhang adapters were appended to the 5′ end of the locus specific primers to accommodate the addition of sample indices via a second PCR step (Forward overhang: 5′-TCGTCGGCAGCGTCAGATGTGTATAAGAGACAG-3′; Reverse overhang: 5′-GTCTCGTGGGCTCGGAGATGTGTATAAGAGACAG-3′). Each 20 μl PCR contained 1X Invitrogen Platinum SuperFi Master Mix (Thermo Fisher Scientific, Waltham, MA, USA), 0.2 µM of each primer, and 0.25 ng μl^−1^ of template. PCR thermal cycling conditions were as follows: initial denaturation at 98 °C for 2 min; followed by 25 cycles of 98 °C for 10 s, 56.5 °C for 20 s, and 72 °C for 15 s; and a final extension at 72 °C for 5 min. No template, mismatched template (fungal DNA), and positive controls were included for all PCRs. PCR was carried out in triplicate for each sample and the reaction products pooled. PCR products were submitted to the Huck Institutes of the Life Sciences (Huck), Genomics Core Facility (The Pennsylvania State University, University Park, PA) where sample indices were added via a second PCR step (10 cycles) using the Illumina Nextera XT Index Kit (Illumina, Inc., San Diego, CA) following the manufacturer’s protocol. Sample libraries were then normalized using a 96-well SequalPrep Normalization Plate Kit (ThermoFisher Scientific, Waltham, MA) following the manufacturer’s protocol. Samples with a normalized concentration of approximately 1.25 ng µl^−1^ were pooled and checked for quality using an Agilent 2100 Bioanalyzer (Agilent, Santa Clara, CA) in conjunction with a High Sensitivity DNA Kit (Agilent, Santa Clara, CA). The final pooled library was quantified using a Kapa Library Quantification Kit (KK4835; Kapa Biosystems, Wilmington, MA) according to the manufacturer’s protocol. The pool was loaded at a final concentration of 7 pM. The pool of libraries was sequenced on an Illumina MiSeq using 250 × 250 paired-end sequencing but utilizing MiSeq Reagent Kit v3 (600 cycle). The raw sequencing reads were deposited in the Sequence Read Archive (SRA) of the National Center for Biotechnology Information (NCBI) database under accession number SRP150539.

### Bioinformatics

Paired-end sequencing data were received in an already de-multiplexed format. Primer sequences were trimmed from the forward and reverse reads using cutadapt [32] before joining the paired-end reads using fastq-join [33] with a minimum overlap of 30 nt and a maximum difference of 30% in the overlap region. Joined reads were then filtered by length to include only those of the expected size (251–256 nt retained). The Quantitative Insights Into Microbial Ecology (QIIME; version 1.8.3) [34] workflow *multiple_split_libraries_fastq.py* was then used to quality filter the remaining reads, retaining reads which were 95% of their original length after truncation at the first base call with a Phred quality score below 20. Quality-filtered sequences were checked for chimeras against the ChimeraSlayer reference dataset (version microbiomeutil-r20110519) using VSEARCH [35].

Downstream analysis of chimera-free quality-filtered sequence sets was carried out using QIIME. Open reference operational taxonomic unit (OTU) clustering using the *pick_open_reference_otus.py* workflow was used to cluster sequences using a combination of de-novo and reference-based methods against the GreenGenes reference database (version 13_8) at 97% sequence similarity. The uclust [36] clustering method was used and only OTUs containing two or more sequences were retained. When using the GreenGenes database to assign taxonomy to 16S rRNA amplicon sequences derived from plant-associated samples, mispriming (and amplification) of plant DNA can be revealed through sequences classified as chloroplast at the class level [37]. All OTUs classified as chloroplast at the class level were filtered from the OTU table using the QIIME script *filter_taxa_from_otu_table.py prior* to diversity analysis and taxonomic summary steps.

Alpha diversity, beta diversity, and taxonomic analysis were performed using the *core_diversity_analysis.py* workflow at a rarefaction depth of 29,500 sequences per sample (other settings default). Additional alpha diversity metrics were calculated using the *alpha_diversity.py* script in QIIME. Principal Coordinate Analysis (PCoA) was carried out on the weighted and unweighted UniFrac distance matrices generated by *core_diversity_metrics.py,* using the *cmdscale* function in base R (version 3.4.4) to produce more suitable plots. To identify differentially abundant taxa between the main treatment groups (acid vs. basic, and mesophilic vs. thermophilic), the OTU table was collapsed to the genus level using the QIIME script *summarize_taxa.py*. The collapsed table was then filtered to exclude genera present in less than 25% of samples and those whose total abundance within the table was less than 150 counts. Filtering was performed using the QIIME script *filter_otus_from_otu_table.py*. Differentially abundant taxa were identified using the QIIME script *group_significance.py,* and comparisons were made using a nonparametric *t* test. The QIIME script *compare_categories.py* was used to analyze the strength and statistical significance of sample groupings (acidic vs. basic and mesophilic vs. thermophilic) in terms of beta diversity. Both weighted and unweighted UniFrac distance matrices were used for comparing groupings under both conditions and the test method was permanova with 999 permutations.

### Statistical analysis

Data are presented as the mean ± standard deviation of triplicate samples. Significant differences between means were tested using one-way analysis of variance (ANOVA) and least significant difference (LSD) tests at a significance level of *p* < 0.05 (Additional file 1: Boxes S1, S2), using Minitab statistical package (Version 3.1, Minitab Inc., USA).

## Results

### Acidogenic digestion performance

**Fig. 1:**
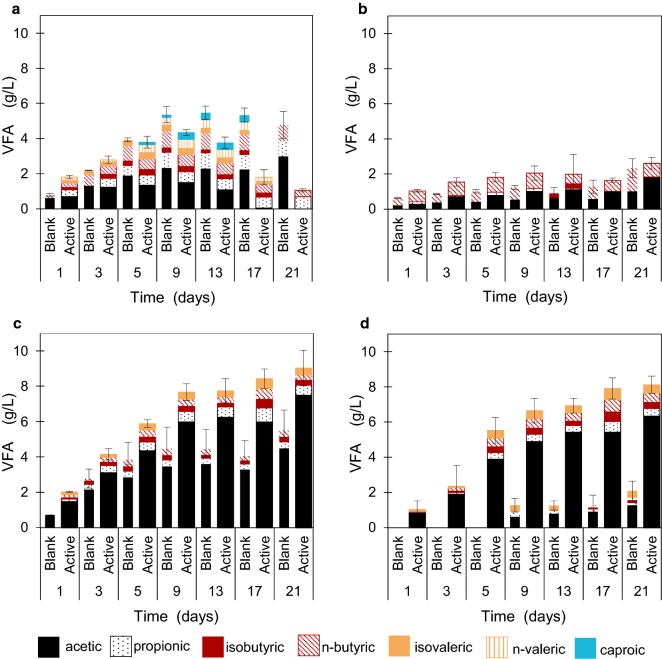
Volatile Fatty Acid profiles of the acidogenic duckweed reactors over 21 days. Reactors were operated under: **a** acidic mesophilic; **b** acidic thermophilic; **c** basic mesophilic; **d** basic thermophilic conditions. Narrow stacked columns represent blank reactors (no inoculum) whereas thick stacked columns represent active (with inoculum) reactors. Error bars are cumulative standard deviations of the individual stacked bars

All reactors produced VFAs, ranging in final concentrations from 1.1 ± 0.1 to 9.0 ± 0.7 mg l^−1^ (Fig. 1). The highest VFA production was observed under basic mesophilic conditions (Fig. 1c), where the average composition consisted of 83.0% acetic, 6.3% propionic, 3.6% isobutyric, 2.7% *n*-butyric, and 4.4% isovaleric acids. These results correspond to a total of 388 ± 28 mg VFA as HAc_eq_ g VS_added_^−1^ (334 ± 24 mg VFA as HAc_eq_ g TS_added_^−1^, Table 1). Approximately, 80% of the final VFA values were achieved by day 13, with an average production rate of 0.59 g HAc_eq_ l^−1^ day^−1^ under these conditions.

**Table 1:** Final volatile fatty acid yields of the blank and active reactors under acidic mesophilic, acidic thermophilic, basic mesophilic, and basic thermophilic conditions

	Acidic mesophilic	Acidic thermophilic	Basic mesophilic	Basic thermophilic
VFA yields (mg VFA as HAc_eq_ g VS_added_^−1^)				
Blank	218 ± 7.7^a^	116 ± 28^ab^	256 ± 37^a^	86 ± 22^b^
Active	55 ± 3.7^a^	117 ± 7.9^b^	388 ± 28^c^	341 ± 2.8^d^

Mean VFA yields were compared separately for blank and active groupings using TUKEY test at a significance level of *p* < 0.05. Superscript letters indicate the resulting statistical groupings within reactor class

**Fig. 2:**
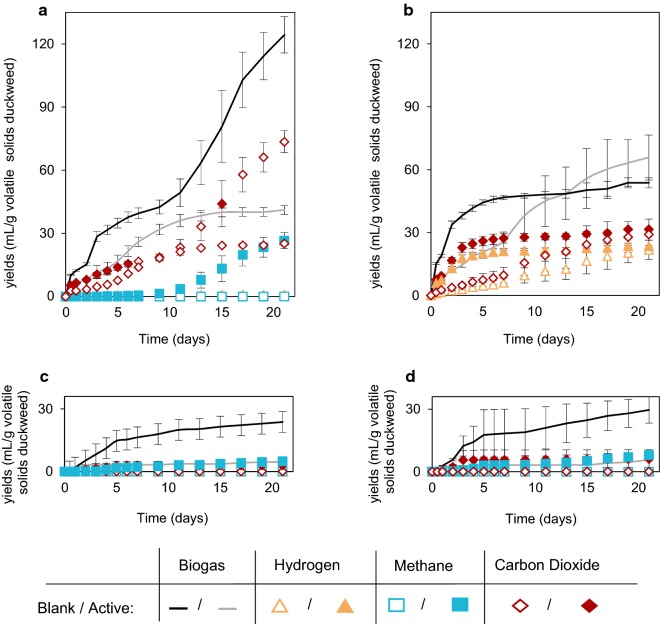
Cumulative biogas, hydrogen, methane, and carbon dioxide yields of the acidogenic duckweed reactors over 21 days. Reactors were operated under: **a** acidic mesophilic; **b** acidic thermophilic; **c** basic mesophilic; **d** basic thermophilic conditions. Blank (no inoculum) reactors are represented as empty bullets whereas active (with inoculum) reactors are represented as solid bullets

The lowest final VFA concentrations were observed in the active reactors operated under acidic mesophilic conditions, in which the acetic acid concentration increased until Day 9 and then gradually disappeared (Fig. 1a), presumably converted into CH_4_ and CO_2_ (Fig. 2a). In order to avoid bias on evaluation of acidogenic digestion performance, it was assumed that the acetate produced had been converted into equal moles of CO_2_ and CH_4_. According to this stoichiometry, the loss in the VFA yield could be back-calculated as 200 ± 20 mg VFA as HAc_eq_ g VS_added_^−1^ (171 ± 17 mg VFA as HAc_eq_ g TS_added_^−1^), in which case the “actual” yield under acidic mesophilic conditions would have been 256 ± 23 mg VFA as HAc_eq_ g VS_added_^−1^ (219 ± 20 mg VFA as HAc_eq_ g TS_added_^−1^).

Among the blank reactors, the final VFA concentrations varied between 2.1 ± 0.5 and 5.9 ± 0.8 mg l^−1^; however, when comparing the yields for all blank reactors, a statistically significant difference was found only between the conditions with the highest (basic mesophilic) and lowest (basic thermophilic) yields (Table 1; Additional file 1: Box S2). In contrast, the final VFA compositions varied between operating conditions (Fig. 1a–c). Potential reasons for these observations are considered in “Discussion”.

Temperature had an adverse effect for blank reactors with no inoculum under basic thermophilic (55 °C) conditions, as their VFA yield of 86 ± 22 mg VFA as HAc_eq_ g VS_added_^−1^ (74 ± 19 mg VFA as HAc_eq_ g TS_added_^−1^) was about one-third of the value observed under mesophilic conditions, observed as 256 ± 37 mg VFA as HAc_eq_ g VS_added_^−1^ (219 ± 32 mg VFA as HAc_eq_ g TS_added_^−1^). The effect of temperature was less pronounced for active reactors operated under basic conditions. Similarly, increased temperature had a negative impact on the average final VFA yield in blank reactors under acidic conditions. Active acidic reactors were more prone to VFA loss due to methanogenic activity; however, the back-calculation of the acetate yields taking the CO_2_ and CH_4_ productions into account shows that the mesophilic (35 °C) conditions would have yielded higher VFA concentrations compared to those of thermophilic conditions for the active reactors as well.

Control reactors without duckweed produced negligible amounts of VFAs, in part because the inocula were pretreated, enriched, and starved prior to the experiments. Also, the substrate–inoculum ratio of 10 used in this study was significantly lower than the common value used for anaerobic digestion trials, which typically varies between 0.5 and 2 for substrates rich in cellulose [38]. Therefore, results pertaining to the effects of endogenous respiration have been omitted.

In both blank and active reactors operated under basic conditions, the acetic acid fraction of VFAs was higher than under acidic conditions, where larger fractions of longer chain VFAs (i.e., propionic, butyric, valeric, caproic) were observed. For instance, under thermophilic conditions, acidic reactors had a final composition of 69.6% acetic and 30.4% butyric acids, whereas basic reactors had a final composition of 78.0% acetic, 5.1% propionic, 4.3% isobutyric, and 6.4% *n*-butyric acids.

Biogas production was observed in all reactors to some extent (Fig. 2); however, the quantities and the compositions varied greatly among treatments. The highest biogas production was recorded in the active acidic reactors operated under mesophilic conditions (124 ± 8.6 ml g duckweed VS_added_^−1^). In these reactors, the predominant gas species recovered was CO_2_ (59.2% of the total gas recovered), followed by CH_4_ (21.3% of the total gas recovered) (Additional file 1: Table S1). The CH_4_ recovery started by Day 9 and reached a cumulative yield of 26.6 ± 3.8 ml g duckweed VS_added_^−1^. High CO_2_ release (61.4% of the total gas recovered) was also observed in the acidic mesophilic blank reactors, but CH_4_ was not produced in the absence of inoculum.

Biogas recovery was minimal in basic reactors under both mesophilic and thermophilic conditions, and was only observed in the first 9 days, mainly as CO_2_ (Fig. 2c, d). Over time, the headspace gas compositions changed and the final contents in active reactors were found to be 1.6 ± 0.04% CO_2_ and 52.5 ± 6.1% CH_4_ in the basic mesophilic reactors, and 2.3 ± 0.3% CO_2_ and 56.8 ± 2.2% CH_4_ in the basic thermophilic reactors. However, significant cumulative recovery of biogas was not observed under either of these conditions.

In contrast to the other three treatments, no CH_4_ was observed under acidic thermophilic conditions. Instead, this was the only condition under which H_2_ was produced (Fig. 2b), with an observed yield of 21.8 ± 4.6 and 23.5 ± 0.5 ml g duckweed VS_added_^−1^ in blank and active reactors, respectively. These values correspond to 33.1% and 43.8% of the total gas recovered from blank to active reactors.

### Carbon balance

**Fig. 3:**
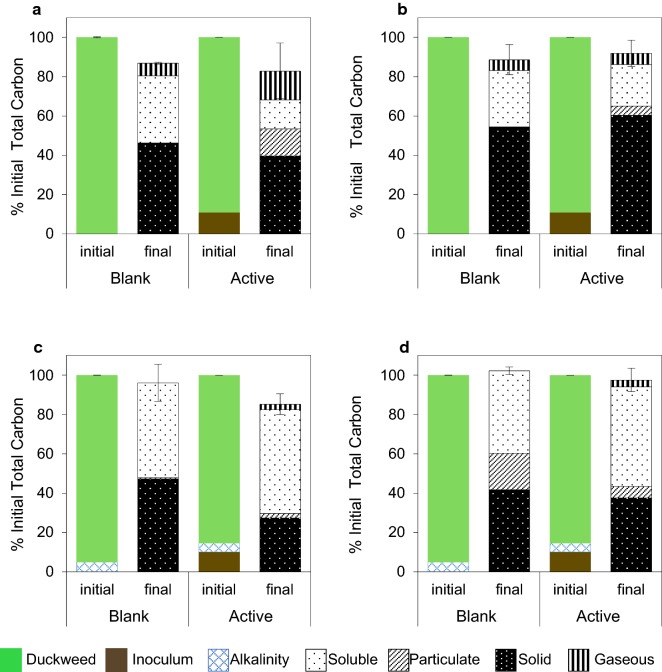
Carbon balance of the acidogenic duckweed reactors. Total carbon percent contributions from initial duckweed, inocula, and alkalinity, and final soluble (< 0.2 µm), particulate (> 0.2 µm; < 340 µm), solid (> 340 µm), and gaseous phases of the reactors under: **a** acidic mesophilic; **b** acidic thermophilic; **c** basic mesophilic; **d** basic thermophilic conditions. Error bars are cumulative standard deviations of the individual measurements

The fractions of initial and final solid, particulate, soluble, and gaseous TC were compared for both blank and active reactors, as a percentage of the initial TC content in each reactor (Fig. 3). Associated chemical oxygen demand balances were reported elsewhere [23]. The average mass closure values on a TC basis varied between 82.9 ± 6.7% and 102.2 ± 1.9% among different operating conditions with and without inoculum addition.

The carbon balance results revealed that the highest solubilization efficiency (i.e., highest increase in the soluble TC content) was achieved under basic mesophilic conditions (52.7%). The lowest final solids content was also observed under these conditions (27.4%). An average of 61.0% of the soluble TC was VFA carbon, accounting for 34.5% of the duckweed TC added in these reactors. The lowest average percentage of soluble TC was found in the acidic mesophilic active reactors; however, the solids were instead converted into particulate and gaseous TC to a higher extent in these reactors compared to others.

The major TC loss to the gaseous phase was observed in the active acidic mesophilic reactors, due to the highest biogas recovery, which consisted of both CO_2_ and CH_4_ (Figs. 2a, 3a). For the rest of the acidic (active and blank) reactors, CO_2_ was the predominant gas. Although not recovered in significant quantities, residual CH_4_ in the reactor headspace constituted most of the TC lost to the gaseous phase in the basic active reactors (Additional file 1: Table S3).

In the acidic blank reactors, the particulate TC concentration was below detection and insignificant compared to the soluble and solid TC values. However, more particulate matter was observed in the active counterparts, supplemented with inoculum. Overall, particulate TC concentration was higher in the basic reactors, with the value observed in basic thermophilic blank reactors (average 18.6%).

Overall, the active reactors exhibited better solids reduction compared to their blank counterparts, except for acidic thermophilic conditions, where the opposite held true (Fig. 3d). In parallel, the final biogas yield was higher in acidic thermophilic blanks, compared to the actives.

### Microbial community analysis

Good’s coverage ranged from 0.957 to 0.999, indicating that a majority of the microbial diversity was captured at the rarefied depth of 29,500 sequences per sample (Table 2). OTU richness varied widely across all samples both in terms of observed OTUs (128–4815) and the chao1 richness estimator (191–6856). Samples with the lowest OTU richness included blank reactors and the active acidic thermophilic reactors. The highest OTU richness was observed for control reactors and inoculum. Samples were ranked similarly with regard to the Simpson diversity index (0.179–0.993) and Shannon diversity index (0.605–9.16), which also account for evenness.

**Table 2:** Alpha diversity metrics for microbial populations in duckweed acidogenically digested under different environmental conditions

Sample type	Good’s coverage	Observed OTUs	Chao1	Shannon diversity index	Simpson diversity index
Acidic mesophilic					
Blank	0.997 ± 0.001	789 ± 86	943 ± 81	4.53 ± 0.21	0.891 ± 0.017
Active	0.990 ± 0.002	1819 ± 82	2495 ± 54	6.04 ± 0.07	0.955 ± 0.003
Control	0.967 ± 0.003	2688 ± 13	3772 ± 240	7.77 ± 0.23	0.974 ± 0.009
Acidic thermophilic					
Blank	0.999 ± 0.000	135 ± 9	221 ± 42	0.85 ± 0.35	0.261 ± 0.117
Active	0.993 ± 0.001	981 ± 89	1511 ± 129	3.64 ± 0.07	0.809 ± 0.018
Control	0.989 ± 0.000	2492 ± 235	2960 ± 258	6.66 ± 0.40	0.945 ± 0.028
Basic mesophilic					
Blank	0.993 ± 0.003	1155 ± 298	1481 ± 381	5.65 ± 0.68	0.947 ± 0.017
Active	0.983 ± 0.002	2145 ± 235	3155 ± 236	6.23 ± 0.41	0.947 ± 0.018
Control	0.960 ± 0.002	4226 ± 98	6141 ± 0	8.61 ± 0.08	0.988 ± 0.002
Basic thermophilic					
Blank	0.993 ± 0.001	1135 ± 151	1463 ± 146	4.62 ± 0.26	0.826 ± 0.025
Active	0.986 ± 0.001	2251 ± 222	3156 ± 361	5.77 ± 0.20	0.916 ± 0.012
Control	0.960 ± 0.004	4626 ± 267	6568 ± 407	9.15 ± 0.01	0.993 ± 0.000
Acidic inoculum	0.976 ± 0.004	2637 ± 242	3706 ± 49	7.12 ± 0.06	0.974 ± 0.001
Basic inoculum	0.961 ± 0.000	3421 ± 92	5129 ± 1	8.48 ± 0.03	0.988 ± 0.000
Duckweed	0.984 ± 0.002	1539 ± 159	2053 ± 129	7.07 ± 0.17	0.971 ± 0.006

**Fig. 4:**
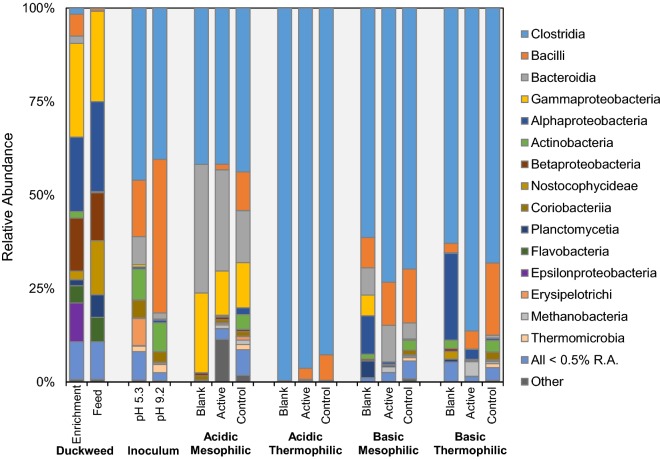
Class-level relative abundance taxonomic bar plot

All reactors were dominated by the class Clostridia, within the phylum Firmicutes, which averaged 70.5% relative abundance (min 35.8%; max 99.6% in blank acidic thermophilic reactors) (Fig. 4). Members of the class Clostridia were rare on duckweed (< 2% relative abundance), but dominant in the inoculum (average 43.3%) suggesting that inoculum mainly contributed to the presence of Clostridia in active and control reactors. However, acidic thermophilic blank reactors were dominated by Clostridia, which is likely due to duckweed-associated Clostridia outcompeting other taxa under these extreme conditions. Other dominant classes of bacteria were: (1) Bacteroidia (phylum Bacteroidetes), present mainly in mesophilic reactors and the acidic inoculum; (2) Gammaproteobacteria (phylum Proteobacteria), present mainly in the acidic mesophilic group (8.5–21.9%), but also prominent on duckweed (average 24.4%); (3) Bacilli (phylum Firmicutes), present in higher abundance in all control reactors, active basic mesophilic reactors, and both acidic and basic inocula, with the highest relative abundance in basic inoculum (42%). The taxonomic profile of duckweed microbes is clearly distinct from both the inocula and the reactors. In addition to Gammaproteobacteria (mentioned above), the dominant bacterial classes associated with duckweed include Alphaproteobacteria (22.2%), which seemed to persist in blank basic reactors (mesophilic and thermophilic), and Betaproteobacteria (13.5%). In addition, Nostocophysideae (phylum Cyanobacteria), Flavobacteriia (phylum Bacteroidetes), and *Epsilonproteobacteria* (phylum Proteobacteria) exhibited moderate relative abundance on duckweed (5–10%), but were low in abundance or absent in reactors. The bacterial class Actinobacteria (phylum Actinobacteria) was present at a moderate relative abundance across inoculum samples (average 8%), but was largely absent from reactors, aside from controls.

The top five genera for each reactor group operated under acidic and basic conditions are given in Tables 3 and 4 respectively, and significant archaeal taxa (relative abundance > 0.01%) are summarized in Table 5. However, taxa outside of the top five may contribute important biochemical pathways (see “Discussion”). In general, the top five genera in reactors accounted for 32.9–99.5% of the observed OTUs (average 63.8%) and the total richness captured by the top five genera showed a strong inverse correlation with alpha diversity metrics, as expected. The top five genera in the inocula were dominated by members of the phylum Firmicutes, while those associated with duckweed were dominated mainly by members of the phylum Proteobacteria. If the top *ten* genera are considered, an additional 10–20% of the OTU richness is described (see Additional file 2 for the relative abundance of all genera in each reactor).

**Table 3:** Relative abundance (R.A.) and cumulative abundance (C.A.) of top five genera in each reactor group operated under acidic conditions

Condition	Type	Taxa	R.A. (%)	C.A. (%)
Acidic mesophilic	Blank	c__Bacteroidia;o__Bacteroidales;f__Prevotellaceae;g__Prevotella	33.1	82.1
		c__Gammaproteobacteria;o__Enterobacteriales;f__Enterobacteriaceae;g__	20.7	
		c__Clostridia;o__Clostridiales;f__Lachnospiraceae;Other	15.2	
		c__Clostridia;o__Clostridiales;f__Ruminococcaceae;g__Ruminococcus	9.1	
		c__Clostridia;o__Clostridiales;f__Veillonellaceae;g__Megasphaera	3.9	
	Active	c__Bacteroidia;o__Bacteroidales;Other;Other	13.4	53.8
		Unassigned;Other;Other;Other;Other;Other	11.4	
		c__Gammaproteobacteria;o__Enterobacteriales;f__Enterobacteriaceae;g__	11.3	
		c__Bacteroidia;o__Bacteroidales;f__Prevotellaceae;g__Prevotella	8.9	
		c__Clostridia;o__Clostridiales;f__Ruminococcaceae;g__Ethanoligenens	8.8	
	Control	c__Gammaproteobacteria;o__Pseudomonadales;f__Pseudomonadaceae;g__Pseudomonas	11.6	32.9
		c__Clostridia;o__Clostridiales;f__Veillonellaceae;g__Acidaminococcus	6.9	
		c__Clostridia;o__Clostridiales;f__Veillonellaceae;g__Succiniclasticum	5.0	
		c__Bacteroidia;o__Bacteroidales;f__Porphyromonadaceae;g__Parabacteroides	4.9	
		c__Bacteroidia;o__Bacteroidales;f__Prevotellaceae;g__Prevotella	4.5	
Acidic thermophilic	Blank	c__Clostridia;o__Clostridiales;f__Clostridiaceae;g__Thermoanaerobacterium	85.5	99.5
		c__Clostridia;o__Clostridiales;f__Clostridiaceae;g__Clostridium	10.0	
		c__Clostridia;o__Clostridiales;f__Ruminococcaceae;g__Ruminococcus	2.5	
		c__Clostridia;o__Clostridiales;f__Ruminococcaceae;g__Ethanoligenens	1.3	
		c__Bacilli;o__Bacillales;f__Planococcaceae;g__	0.1	
	Active	c__Clostridia;o__Clostridiales;f__Ruminococcaceae;g__Ethanoligenens	43.0	90.6
		c__Clostridia;o__Clostridiales;f__Clostridiaceae;g__Thermoanaerobacterium	20.6	
		c__Clostridia;o__Clostridiales;f__Ruminococcaceae;g__Ruminococcus	20.6	
		c__Clostridia;o__Clostridiales;f__Clostridiaceae;g__Clostridium	4.0	
		c__Clostridia;o__Clostridiales;f__Lachnospiraceae;g__Coprococcus	2.4	
	Control	c__Clostridia;o__OPB54;f__;g__	20.0	59.9
		c__Clostridia;o__SHA-98;f__D2;g__	11.2	
		c__Clostridia;o__;f__;g__	11.0	
		c__Clostridia;o__Clostridiales;f__Clostridiaceae;g__Caloramator	9.1	
		c__Clostridia;o__SHA-98;f__;g__	8.6

**Table 4:** Relative abundance (R.A.) and cumulative abundance (C.A.) of top five genera in each reactor group operated under basic conditions

Condition	Type	Taxa	R.A. (%)	C.A. (%)
Basic mesophilic	Blank	c__Clostridia;o__Clostridiales;f__Clostridiaceae;g__Alkaliphilus	14.1	48.8
		c__Clostridia;o__Clostridiales;f__Lachnospiraceae;g__Coprococcus	13.0	
		c__Clostridia;o__Clostridiales;f__Lachnospiraceae;g__	7.5	
		c__Bacteroidia;o__Bacteroidales;f__Porphyromonadaceae;g__	7.3	
		c__Clostridia;o__MBA08;f__;g__	6.9	
	Active	c__Clostridia;o__MBA08;f__;g__	23.9	66.7
		c__Clostridia;o__Clostridiales;f__Ruminococcaceae;g__	19.2	
		c__Bacilli;o__Bacillales;f__Bacillaceae;g__Natronobacillus	9.6	
		c__Bacteroidia;o__Bacteroidales;f__Porphyromonadaceae;g__	8.6	
		c__Clostridia;o__Clostridiales;f__Lachnospiraceae;g__	5.4	
	Control	c__Clostridia;o__MBA08;f__;g__	25.9	43.9
		c__Bacilli;o__Bacillales;f__;g__	6.2	
		c__Clostridia;o__Clostridiales;f__Lachnospiraceae;g__	4.8	
		c__Clostridia;o__Clostridiales;f__;g__	3.6	
		c__Clostridia;o__Clostridiales;f__Ruminococcaceae;g__	3.5	
Basic thermophilic	Blank	c__Clostridia;o__Clostridiales;f__Caldicoprobacteraceae;g__Caldicoprobacter	45.1	80.2
		c__Clostridia;o__Clostridiales;f__[Tissierellaceae];g__Tepidimicrobium	17.6	
		c__Alphaproteobacteria;o__Rhodobacterales;f__Rhodobacteraceae;g__Rhodobacter	7.5	
		c__Alphaproteobacteria;o__Rhizobiales;f__Rhizobiaceae;g__Agrobacterium	6.8	
		c__Acidimicrobiia;o__Acidimicrobiales;f__C111;g__	3.3	
	Active	c__Clostridia;o__Clostridiales;f__Caldicoprobacteraceae;g__Caldicoprobacter	24.5	73.2
		c__Clostridia;o__Halanaerobiales;f__Halanaerobiaceae;g__	19.1	
		c__Clostridia;o__OPB54;f__;g__	13.6	
		c__Clostridia;o__MBA08;f__;g__	12.4	
		c_Methanobacteria;o_Methanobacteriales;f_Methanobacteriaceae;g__Methanothermobacter	3.6	
	Control	c__Clostridia;o__MBA08;f__;g__	10.8	33.7
		c__Clostridia;o__Clostridiales;f__Lachnospiraceae;g__	8.0	
		c__Bacilli;o__Bacillales;f__;g__	6.6	
		c__Clostridia;o__Clostridiales;f__Ruminococcaceae;g__	4.1	
		c__Clostridia;o__Clostridiales;f__Lachnospiraceae;g__Butyrivibrio	4.1

**Table 5:** Relative abundance (R.A.) and cumulative abundance (C.A.) of top five archaeal genera in each reactor group

Condition	Type	Taxa	R.A. (%)	C.A. (%)
Acidic mesophilic	Blank	None		None
	Active	c__Methanomicrobia;o__Methanosarcinales;f__Methanosarcinaceae;g__Methanosarcina	2.03	3.03
		c__Methanobacteria;o__Methanobacteriales;f__Methanobacteriaceae;g__Methanobrevibacter	0.83	
		c__Methanobacteria;o__Methanobacteriales;f__Methanobacteriaceae;g__Methanobacterium	0.13	
		c__Thermoplasmata;o__E2;f__[Methanomassiliicoccaceae];g__vadinCA11	0.04	
	Control	c__Thermoplasmata;o__E2;f__[Methanomassiliicoccaceae];g__vadinCA11	0.77	2.35
		c__Methanobacteria;o__Methanobacteriales;f__Methanobacteriaceae;g__Methanobacterium	0.69	
		c__Methanobacteria;o__Methanobacteriales;f__Methanobacteriaceae;g__Methanobrevibacter	0.45	
		c__Methanomicrobia;o__Methanosarcinales;f__Methanosarcinaceae;g__Methanosarcina	0.19	
		c__Thaumarchaeota;o__Nitrososphaerales;f__Nitrososphaeraceae;g__Candidatus Nitrososphaera	0.16	
Acidic thermophilic	Blank	None		< 0.1
	Active	c__Methanomicrobia;o__Methanosarcinales;f__Methanosarcinaceae;g__Methanosarcina	0.03	< 0.1
	Control	c__Methanomicrobia;o__Methanosarcinales;f__Methanosarcinaceae;g__Methanosarcina	0.02	< 0.1
Basic mesophilic	Blank	c__MCG;o__pGrfC26;f__;g__	0.02	< 0.1
	Active	c__Methanobacteria;o__Methanobacteriales;f__Methanobacteriaceae;g__Methanobrevibacter	1.45	1.49
		c__Thermoplasmata;o__E2;f__[Methanomassiliicoccaceae];g__Methanomassiliicoccus	0.02	
		c__Methanobacteria;o__Methanobacteriales;f__Methanobacteriaceae;g__Methanobacterium	0.01	
	Control	c__Methanobacteria;o__Methanobacteriales;f__Methanobacteriaceae;g__Methanobrevibacter	0.35	0.73
		c__Thermoplasmata;o__E2;f__[Methanomassiliicoccaceae];g__Methanomassiliicoccus	0.14	
		c__Thaumarchaeota;o__Nitrososphaerales;f__Nitrososphaeraceae;g__Candidatus Nitrososphaera	0.13	
		c__Methanomicrobia;o__Methanosarcinales;f__Methanosarcinaceae;g__Methanosarcina	0.05	
		c__Methanobacteria;o__Methanobacteriales;f__Methanobacteriaceae;g__Methanosphaera	0.0	
Basic thermophilic	Blank	None	0.00	< 0.1
	Active	c__Methanobacteria;o__Methanobacteriales;f__Methanobacteriaceae;g__Methanothermobacter	3.58	3.93
		c__Methanobacteria;o__Methanobacteriales;f__Methanobacteriaceae;g__Methanobacterium	0.28	
		c__Thermoplasmata;o__E2;f__[Methanomassiliicoccaceae];g__Methanomassiliicoccus	0.02	
		c__Methanobacteria;o__Methanobacteriales;f__Methanobacteriaceae;g__Methanobrevibacter	0.02	
		c__Thaumarchaeota;o__Nitrososphaerales;f__Nitrososphaeraceae;g__Candidatus Nitrososphaera	0.01	
	Control	c__Methanobacteria;o__Methanobacteriales;f__Methanobacteriaceae;g__Methanobrevibacter	0.27	0.97
		c__Thermoplasmata;o__E2;f__[Methanomassiliicoccaceae];g__Methanomassiliicoccus	0.24	
		c__Methanobacteria;o__Methanobacteriales;f__Methanobacteriaceae;g__Methanothermobacter	0.15	
		c__Thaumarchaeota;o__Nitrososphaerales;f__Nitrososphaeraceae;g__Candidatus Nitrososphaera	0.12	
		c__Methanomicrobia;o__Methanosarcinales;f__Methanosarcinaceae;g__Methanosarcina	0.10	

Archaea were absent from the top five genera in all reactors except the active basic thermophilic reactors, which contained 3.6% *Methanobacteriaceae methanothermobacter.* Overall, the archaeal content of the reactors was low, ranging from none detected up to approximately 4% relative abundance in the active basic thermophilic reactors. Other dominant archaea (> 1% relative abundance) included *Methanosarcinaceae methanosarcina* (2% in active acidic mesophilic reactors) and *Methanobacteriaceae methanobrevibacter* (1.5% in active basic mesophilic reactors). In general, all acidic thermophilic reactors, blanks from all conditions, and duckweed samples exhibited negligible fractions of archaea.

## Discussion

### Effect of pH and temperature on acidogenic digestion performance

The experiments revealed high variations in VFA production potentials at different pH and temperature values. The highest VFA yield observed 388 ± 28 mg VFA as HAc_eq_ g VS_added_^−1^ (332 ± 24 mg VFA as HAc_eq_ g TS_added_^−1^) under basic mesophilic conditions is similar to the findings of a study conducted by Yuan et al. [39] on acidogenic digestion of activated wastewater sludge at pH 10 and ambient temperature. The authors reported 233 mg VFA as HAc_eq_ g VS^−1^, attributing the high performance to the availability of soluble proteins under these conditions. The superior performance achieved in our study might be due to the high carbohydrate content of duckweed biomass, in addition to proteins. In parallel, basic mesophilic conditions resulted in high acetic acid content (up to 83% of total VFAs). Apart from its effect on protein solubilization, high pH also has a chemical pretreatment effect on cellulosic and hemicellulosic biomass, causing the release of acetyl groups, which could explain the high acetic acid concentrations observed under these conditions. The same effect has also been reported by other researchers for the acidogenic digestion of food waste at elevated pH [40]. The relative effects of biotic and abiotic conversion mechanisms on high acetic acid yields are further discussed below in this section.

In addition, H_2_ recovery observed under acidic thermophilic conditions (up to 23.5 ± 0.5 ml g duckweed VS_added_^−1^) was comparable to a study on swine wastewater-derived duckweed (*Lemna minor*) mesophilic fermentation to biohydrogen, which resulted in 13 ml H_2_ g^−1^ dry duckweed for non-pretreated biomass [41]. The higher values observed in our study could be due to thermophilic conditions. In the same study, the researchers reported up to 42% H_2_ content, which was also in agreement with our findings of 33.1–43.8%. These results are also within the range of specific H_2_ production potentials of materials characteristic of the organic fraction of municipal solid waste, such as cabbage, carrot, and rice, reported as 19.3–96.0 ml H_2_ g VS^−1^ with 27.7–55.1% H_2_ content [42].

Overall, although the acetate produced under acidic mesophilic conditions was lost in the form of CH_4_, the mesophilic reactors produced more VFAs than the thermophilic reactors in both acidic and basic reactors, with and without inoculum supplementation. As observed for activated sludge by Yu et al. [15], the present study with duckweed also found that pH has a more significant impact than temperature on VFA production. Yu et al. attributed this observation to enhanced substrate availability due to chemical hydrolysis under alkaline conditions at both mesophilic and thermophilic temperatures [15]. However, our observation might also be due to the presence of alkaliphilic thermophiles originating from compost to the absence of acidophilic thermophiles in the enriched inoculum mixture.

### Effect of operating conditions on microbial community diversity and composition

#### Alpha diversity

Within each tested condition, blank reactors without inoculum were found to be less diverse than active reactors, which were in turn less diverse than control reactors without duckweed (Table 2). The lack of diversity in blank reactors is likely due to the fact that the sole source of microbes in these reactors was from duckweed, which was harvested from an aerobic environment. These aerobic microbes, introduced into an anaerobic environment, are not expected to flourish. In general, the diversity of blank basic reactors (both mesophilic and thermophilic) was similar to, but slightly lower than, the diversity of the duckweed microbes, while acidic conditions (especially thermophilic) led to a decrease in the diversity in those blank reactors. Controls had the highest alpha diversity within each treatment group and were generally similar to the inoculum for acidic control reactors, but diversity slightly *increased* from the basic inoculum to the basic controls. Since inoculum was the sole source of microbes in the control reactors, it is reasonable that the diversity would be similar, but the reasons for the slight increase in diversity observed in the basic controls are unclear. In active reactors, the decrease in diversity from the inoculum (presumably the major source of microbes in active reactors) is reasonable given the potential selective pressures of an active microbial community in the presence of substrate (duckweed biomass). In general, diversity increased among the active reactors as follows: acidic thermophilic ≪ acidic mesophilic < basic mesophilic ≈ basic thermophilic. The very low diversity in acidic thermophilic reactors is reasonable given the extreme conditions present there. Low diversity has previously been noted for thermophilic cultures [43]. Similar trends were observed for blank and control reactors across treatment groups with respect to all diversity measures, except for acidic thermophilic controls, which suffered less diversity loss in relation to acidic mesophilic conditions than their blank and active counterparts.

#### Beta diversity

**Fig. 5:**
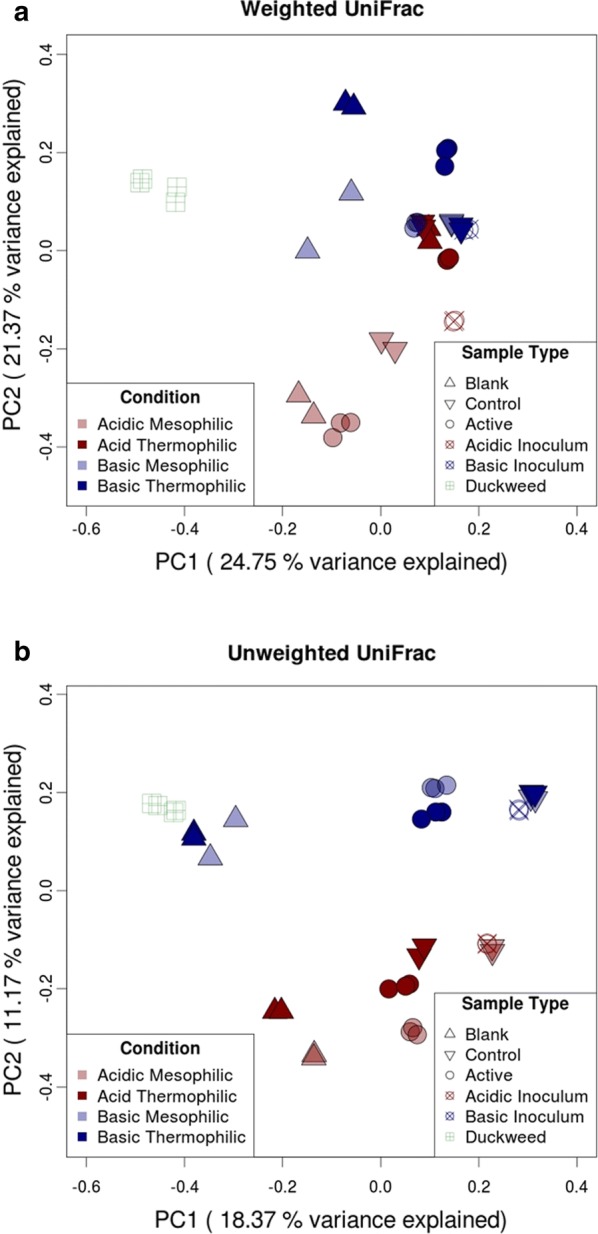
**a** Weighted and **b** unweighted PCoA plots

Principle coordinate analysis (PCoA) using both abundance-weighted and unweighted UniFrac distances showed reasonable clustering effects (Fig. 5a, b). All replicates clustered closely together except blank basic mesophilic replicates, which were still reasonably associated. PCoA of weighted UniFrac distance explained more of the variation (PC1—24.75% and PC2—21.37%) compared to unweighted distances (PC1—18.37% and PC2—11.17%); however, unweighted UniFrac PCoA clustered very clearly according to sample group. In the unweighted PCoA plot, the most prominent clustering effect is by pH regime (PC2), with duckweed samples clustering with all basic samples. PC1 appears to separate the samples based on sample type (blank, active, control). Blank reactors without inoculum are clearly more similar to duckweed samples, and control samples without duckweed cluster very tightly with the inoculum, which was the only source of microbes in these reactors. Acidic thermophilic controls diverge somewhat from the acidic inoculum. Comparing active reactors, it appears that temperature had a greater effect on differentiating acidic reactors than basic reactors (degree of separation, PC2). The same appears to be true for blanks.

The weighted PCoA plot still shows significant clustering by pH regime; however, all acidic thermophilic reactors appear to cluster more closely with basic inoculum, active basic mesophilic reactors, and basic controls. The reasons for this are unclear. The weighted PCoA plot also shows a greater degree of separation between acidic mesophilic and acidic thermophilic reactors than does the unweighted plot, and duckweed appears to be more distinct from blank reactors on a weighted basis. In the literature, it has been noted that qualitative measures such as unweighted UniFrac distances better reveal the effect of different founding populations and the ability of microbes to survive under different conditions, while quantitative measures (weighted UniFrac) better show the effect of transient factors (e.g., nutrient availability) [44]. Here, the weighted PCoA analysis does not seem to reflect the various VFA profiles as well as the unweighted PCoA. Statistical analysis of sample groupings (acidic vs. basic, and mesophilic vs. thermophilic) confirmed the significance of these groupings. Analysis based on weighted UniFrac distances revealed statistical significance for both pH and temperature groupings (*p* value 0.001); however, the test statistic for the pH grouping was slightly higher (7.01 vs. 6.11), indicating a stronger effect. Grouping by temperature was significant on the basis of unweighted UniFrac distances as well, but to a lesser degree than with weighted distances (*p* value 0.009; test statistic 1.94), while pH grouping was deemed to be very significant under both measures (unweighted *p* value 0.001; test statistic 3.81). These results back up the clustering observed in the PCoA plots and indicate that pH had a stronger effect in determining the microbial community composition (both qualitatively and quantitatively) than did temperature, for the conditions tested.

#### Composition

Analysis of differential abundance at the genus level (acidic vs. basic, and mesophilic vs. thermophilic) was performed using reactor samples only (i.e., no inoculum or duckweed samples) and revealed a greater number of differentially abundant taxa across pH regimes than across temperature regimes (71 vs. 22 based on FDR-corrected *p* values < 0.05). See Additional file 3 for complete results of the comparison.

Of the differentially abundant taxa across temperature regimes, only five were enriched in thermophilic reactors and all were members of the phylum Firmicutes. These included *Clostridiaceae thermoanaerobacterium*, *Tissierellaceae tepidimicrobium*, *Planococcaceae lysinibacillus*, and *Thermoanaerobacterales thermovenabulum*, along with unidentified members of the order OPB54. The percent difference in the mean counts of these genera between the two conditions exceeded 80% in each case (average 95%), indicating that temperature was strongly selective for these microbes. On the other hand, 17 of the genera with temperature-dependent differential abundance were enriched in mesophilic reactors (Additional file 3). Among those with the largest increase in observed counts under mesophilic conditions were *Prevotellaceae prevotella,* unidentified genera in the families *Enterobacteriaceae* and *Porphyromonadaceae*, and unidentified members of the order Bacteroidales (percent difference in mean counts > 99%). Only one member of the kingdom archaea was differentially abundant across temperature regimes (*Methanobacteriaceae methanobrevibacter*), preferring mesophilic conditions.

Differentially abundant taxa across pH regimes are too numerous to detail (Additional file 3), but some key taxa that support the validity of the differential abundance analysis include the enrichment of *Veillonellaceae acidaminococcus* in acidic samples, and *Clostridiaceae alkaliphilus* in the basic samples. In fact, nearly half of the 14 genera enriched in the acidic samples belong to the family *Veillonellaceae*. Others belong mostly to the class Clostridia, with two examples from the class Bacteroidetes. The remaining 57 genera fared better under basic conditions. Some were only moderately enriched under basic conditions (e.g., unidentified genus in the family *Lachnospiraceae*; 71% difference in mean counts), while others were completely absent from acidic reactors—*Bacillaceae natronobacillus*, *Clostridiaceae natronincola_anaerovirgula*, *and Bacteroidaceae bacteroides,* for example. Overall, phyla enriched in basic reactors were more diverse, including Firmicutes, Bacteroidetes, Tenericutes, Actinobacteria, Cyanobacteria, and Proteobacteria. Only two genera of archaea were found be differentially abundant across pH regimes, both preferring basic conditions—*Methanomassiliicoccaceae methanomassiliicoccus* and *Methanobacteriaceae methanothermobacter.*

#### Relationships between operating conditions, microbial community structure, and end products

The differences in operational parameters of the reactors provided unique environments which led to distinct microbial communities and the production of different end products under each condition (Figs. 1, 2, 3, 4). The effects of pH and temperature on microbial populations and end-product profiles during acidogenic digestion of duckweed have been summarized in Table 6. For example, the genus *Acidaminococcus,* a mesophilic anaerobic gram-negative cocci which can ferment amino acids [45], was observed only in acidic mesophilic reactors. *Thermoanaerobacterium*, a genus with members which can degrade starch, cellulose, and sucrose for H_2_ production, favors slightly acidic conditions [46], and was observed here as one of the most dominant genera under acidic thermophilic conditions. In contrast, basic conditions were dominated by cultures originating from alkaline environments. For instance, *Natronobacillus*, a genus of alkaliphile anaerobic species with the capability to fix nitrogen [47], was identified in the basic mesophilic reactors. The negative gas pressure reported in these reactors (Additional file 1: Table S4) may have been caused by the fixation of the nitrogen gas by these organisms. Another family of bacteria which was abundant in basic mesophilic reactors, Porphyromonadaceae, has been previously isolated from mesophilic anaerobic reactors [50]. In addition, some uncultivated bacterial lineages such as MBA08 [Clostridia] and OPB54 [Clostridia] which were previously detected in anaerobic digesters, were present in the basic reactors tested here. *Tepidimicrobium*, a xylanolytic genus with thermophilic and alkali-tolerant members [48], was detected under basic thermophilic conditions, along with *Halanaerobiacea*, a thermophilic genus found in agricultural biogas plants [49]. Some genera, such as *Coprococcus, Ethanoligenens,* and *Clostridium* were observed under both acidic and basic conditions.

**Table 6:** Summary of microbial populations and end product profiles under various operating conditions

Conditions	Key findings
Acidic mesophilic	Susceptible to VFA loss due to acetoclastic methanogenic activity (*Methanosarcina*, 2.03%) High biogas-CO_2_ content, suggesting fast hydrolysis, resulting in TAN release Low CH_4_ yield (26.6 ± 3.8 ml g duckweed VS_added_^−1^) compared to literature, likely because the very high ammonium concentrations required as buffer were inhibitory
Acidic thermophilic	H_2_ recovery up to 23.5 ± 0.5 ml g^−1^ duckweed solids added Least diverse microbial communities (α diversity) Acetate and butyrate were predominant VFA species
Basic mesophilic	Highest VFA yields (388 ± 28 mg VFA as HAc_eq_ g VS_added_^−1^) Competition between homoacetogenesis and hydrogenotrophic methanogenesis over H_2_ Low biogas recovery (23.7 ± 6.2 ml g duckweed VS_added_^−1^) compared to literature, suggesting presence of internal sinks for headspace H_2_ and CO_2_
Basic thermophilic	Highest final particulate matter formation (18.6% of initial total carbon) in the absence of inoculum, suggesting chemical (alkaline) pretreatment augmented VFA production Low biogas recovery (29.7 ± 6.3 ml g duckweed VS_added_^−1^), suggesting presence of internal sinks for headspace H_2_ and CO_2_
Overall conclusions	Within 9 days, more than 80% of the final day VFA concentrations were achieved Species richness (α diversity) was higher in basic reactors pH has a more significant impact than temperature on both the composition of microbial communities (β diversity) and VFA production

The high acetic acid yields observed under basic conditions were very likely augmented by homoacetogenesis. The presence of hydrolytic and fermentative taxa such as the families Porphyromonadaceae [50] and Ruminococcaceae [51], and the genera *Prevotella* [52], and *Caldiocoprobacter* [50], might have theoretically resulted in the production H_2_ and CO_2_. In contrast, the biogas recovery observed was negligible (24 ml/g duckweed VS_added_), which suggests that the produced H_2_ and CO_2_ might be converted into acetate by homoacetogenic bacteria. While it is not possible to positively identify homoacetogenic species given the resolution of the current data set, taxonomic groups which are known to contain homoacetogens were abundant in the basic thermophilic reactors. These include the genus *Clostridium* (2.8% relative abundance), within which thermophilic homoacetogenic species have been identified in the literature (e.g., *C. thermoaceticum* and *C. thermoautotrophicum*), and the order Thermoanaerobacterales (4.5% relative abundance), which is known to encompass thermophilic homoacetogens of the genus *Thermoanaerobacter* (e.g., *T. kivui*) [53, 54]. The negative headspace pressures recorded in both mesophilic and thermophilic reactors at pH 9 also support this conclusion (Additional file 1: Table S4), and indicate that homoacetogens are not inhibited at pH 9. However, an evolution of CH_4_ was also observed in the headspace, especially after Day 5, where no biogas was recovered, but rather the headspace H_2_ and CO_2_ contents decreased. In both basic mesophilic and basic thermophilic reactors, hydrogenotrophic methanogenesis was observed, potentially due to the activity of genera such as *Methanobrevibacter*, which was also reported by Gaby et el. [43] in anaerobic digesters fed with food waste. However, the absence of acetotrophic genera such as *Methanosarcina*, along with high acetate concentrations, shows that at pH 9, neither 35 °C nor 55 °C favored acetoclastic methanogens.

Although it was previously reported that methanogenic activity could be inhibited under pH 6 [55], the reduction of acetate and generation of CH_4_ under mesophilic conditions here revealed acetoclastic methanogenic activity, which is likely related to the presence of *Methanosarcina* sp. *Methanosarcina* are capable of both acetoclastic and hydrogenotrophic methanogenesis (Table 4), so this finding could also explain the absence of H_2_ in the headspace. This outcome might be a result of effective solids reduction and hydrolysis (Fig. 3a), leading to high protein degradation and subsequent ammonium release, which provided local pH increases and buffering capacity, thereby creating a suitable environment for methanogens. In fact, others have similarly reported that co-fermentation of food waste and excess sludge provided favorable conditions for high solubilization, leading to higher ammonia concentrations and slight VFA loss to CH_4_ during acidogenic digestion [56]. The present results also indicate that methanogenic activity could not be permanently inhibited by heat pretreatment, similar to the findings of Luo et al. [55]. However, the biomethane recovery observed in this study (Fig. 2a, 26.6 ± 3.8 ml g duckweed VS_added_^−1^) was not comparable to biochemical methane potential studies of raw duckweed reported in the literature, which were 158 ml g VS_added_^−1^ from *Lemna minor* [57] and 259 ml g VS_added_^−1^ [23] from *Lemna obscura*. This could be primarily because of the tenfold higher substrate–inoculum ratio provided in the present study, which may have caused simultaneous substrate inhibition due to ammonia and VFA accumulation [58]. These conditions might have led to an inhibited state at which the process was stable, but yielded lower CH_4_ [59]. In fact, the free ammonia concentrations reported in this study (Additional file 1: Table S2) have been previously reported to have potential inhibitory effects [60]. In addition, the higher CO_2_ recovery observed in this study could be due to the activity of syntrophic bacterial populations (producing CO_2_ and H_2_ from acetate), such as some members of *Coprococcus* and *Clostridium* [61], which also might have acted as a sink for acetate.

In contrast to acidic mesophilic conditions, acidic thermophilic conditions may have inhibited methanogenic activity, potentially due to lower solids solubilization efficiency (Fig. 3b). This may be why lower ammonia concentrations were observed under acidic thermophilic conditions (Additional file 1: Table S2), and local increases in pH were not favored. This is consistent with the literature: methanogenic activity is known to be more easily suppressed under thermophilic conditions in the presence of lower ammonia concentrations [59]. Furthermore, the acidic thermophilic reactors were heavily dominated with genera containing H_2_-forming members such as *Ethanoligenens* [62] and *Clostridium* [63]; as well as *Ruminococcus* [64] and *Thermoanaerobacterium* [46], which include sugar-fermenting thermophiles that can produce acetate and butyrate (Fig. 1b).

The activity originating from the microorganisms associated with duckweed may have significantly affected solubilization of the biomass and its conversion into VFAs (Fig. 3), as VFA production was also observed in blank reactors containing duckweed to which no inoculum was provided. This suggests that the biofilm present on duckweed can serve as suitable environment for anaerobic microorganisms. The VFA production was higher in mesophilic blank reactors, compared to those operated under thermophilic conditions. This might be because the mesophilic operating conditions are closer to the natural habitat of duckweed. Under acidic thermophilic conditions, the blanks were dominated by spore-forming bacteria, which might have survived in the natural habitat of duckweed biofilm until favorable conditions prevailed. In most cases, addition of inoculum resulted in higher reactor performance in terms of solubilization and VFA production. Only in acidic thermophilic reactors, better solubilization efficiency was observed for blanks (with no inoculum); however, the VFA yields were still slightly lower than in the actives. The lowest VFA production performance was observed in blank reactors under basic thermophilic conditions. This suggests that under basic conditions, VFA production was mainly carried out through biotic processes, rather than as an effect of chemical pretreatment releasing acetyl groups from hemicellulose [65], as has been previously observed during alkaline pretreatment of cellulosic biomass [66].This finding was further supported by relatively higher chemical oxyden demand (i.e. less biological degradation) of the basic reactor solids, as reported previously [23]. However, another interesting point to note in the thermophilic blank reactors is the increase in the particulate matter fraction. The particulates were only evident in basic blank reactors, and were markedly higher in concentration under thermophilic conditions. This may imply that the basic conditions augmented acetate production by a chemical pretreatment effect, which increased the efficiency of hydrolysis and in turn increased the bioavailability of the biomass for microbial conversion. Overall, the results indicate that the enhanced VFA production observed under basic conditions was an outcome of a synergy between chemical pretreatment and biological activity.

## Conclusions

This study demonstrated that 33.2 ± 2.4% of duckweed biomass can be converted into VFAs with a mixed culture microbiome under basic mesophilic conditions. The superior performance observed under these conditions was attributed to both chemical treatment and microbial bioconversion. Final yield and composition of the VFAs primarily depended on the pH and much less on the temperature of the reactors. The composition of the microbial community under these different conditions was also affected more by pH than temperature, with temperature effects enhanced under acidic conditions as compared to basic conditions. Depending on the end product of interest, pH can be adjusted either to produce longer chain VFAs and H_2_ (under acidic conditions), or to maximize total VFA yields (under basic conditions). VFAs can be further processed into medium chain fatty acids, which are building blocks for high-value advanced biofuels. Avoidance of the pH window which favors methanogenic activity during acidogenic digestion would enable downstream processing of carboxylic acid production residuals through methanogenic anaerobic digestion to maximize energy recovery.

These results indicate that duckweed is a technically feasible alternative feedstock for the production of advanced biofuel precursors. In addition, the residual biomass from the VFA production process could be valorized through conversion into biogas and biosolids. To more completely access the feasibility of this process, studies on the conversion of duckweed into multiple end products in a complete biorefinery system are necessary.

## Additional files

**Additional file 1.** Additional data related to chemical analysis; this file contains: final headspace and overall recovered biogas volumetric compositions in reactors at final time point; Total ammonifiable nitrogen and associated ammonium and ammonia concentrations in reactors at final time point; Carbon balance details of reactors at initial and final time points; Headspace pressure in reactors over time; and One-way ANOVA and TUKEY comparison results of VFA yields achieved in active and blank reactors.

**Additional file 2.** Genus level relative abundance; this file contains the relative abundance of each genus in each sequenced sample in the form of a genus × sample matrix.

**Additional file 3.** Differential abundance analysis; this file contains the complete results of the differential abundance analysis comparing the abundance of genera between pH conditions (acidic vs. basic) and temperature conditions (mesophilic vs. thermophilic).
