# Supplementary material for: Effect of pH and temperature on microbial community structure and carboxylic acid yield during the acidogenic digestion of duckweed
Source: Biotechnol Biofuels. 2018 Oct 8;11:275. doi: 10.1186/s13068-018-1278-6 (PMC6174553; doi:10.1186/s13068-018-1278-6)
Supplement: Supplementary file 1 — Additional file 1. Additional data related to chemical analysis; this file contains: final headspace and overall recovered biogas volumetric compositions in reactors at final time point; Total ammonifiable nitrogen and associated ammonium and ammonia concentrations in reactors at final time point; Carbon balance details of reactors at initial and final time points; Headspace pressure in reactors over time; and One-way ANOVA and TUKEY comparison results of VFA yields achieved in active and blank reactors. [file 13068_2018_1278_MOESM1_ESM.docx]

**EFFECT OF PH AND TEMPERATURE ON MICROBIAL COMMUNITY STRUCTURE AND CARBOXYLIC ACID YIELD DURING THE ACIDOGENIC DIGESTION OF DUCKWEED**

**Additional File 1**

**Table S1:** Final headspace and overall recovered biogas volumetric compositions in reactors at final time point

| Reactors: | Final Day Headspace gas composition (%) | | | | Overall composition of recovered biogas (%) | | | |
| --- | --- | --- | --- | --- | --- | --- | --- | --- |
|  | **Hydrogen** | **Methane** | **Carbon Dioxide** | **Hydrogen** | | **Methane** | **Carbon Dioxide** |  |
| BAM1 | 6.70 | 0.00 | 84.10 | 2.77 | | 0.03 | 56.32 |  |
| BAM2 | 10.27 | 0.45 | 81.17 | 4.56 | | 0.02 | 66.39 |  |
| AM1 | 0.00 | 32.73 | 71.93 | 0.58 | | 19.74 | 56.95 |  |
| AM2 | 2.57 | 31.11 | 73.68 | 0.63 | | 23.36 | 58.85 |  |
| AM3 | 0.00 | 30.99 | 73.47 | 0.80 | | 20.67 | 61.67 |  |
| CAM1 | 0.00 | 1.51 | 3.53 | 0.00 | | 0.02 | 1.46 |  |
| CAM2 | 0.00 | 1.20 | 3.06 | 0.00 | | 0.05 | 1.77 |  |
| BAT1 | 54.95 | 0.00 | 51.80 | 39.16 | | 0.00 | 47.62 |  |
| BAT2 | 53.70 | 0.00 | 46.93 | 34.24 | | 0.00 | 39.55 |  |
| AT1 | 45.58 | 0.00 | 59.16 | 44.07 | | 0.00 | 57.97 |  |
| AT2 | 9.02 | 0.00 | 68.02 | 45.53 | | 0.00 | 63.04 |  |
| AT3 | 25.20 | 0.00 | 70.79 | 42.75 | | 0.00 | 55.38 |  |
| CAT1 | 1.37 | 0.00 | 4.30 | 0.00 | | 0.00 | 3.12 |  |
| CAT2 | 0.00 | 0.00 | 3.80 | 0.00 | | 0.00 | 2.76 |  |
| BBM1 | 0.00 | 0.00 | 1.35 | 0.00 | | 0.00 | 1.37 |  |
| BBM2 | 0.00 | 0.00 | 1.30 | 0.00 | | 0.00 | 1.31 |  |
| BM1 | 0.00 | 47.10 | 1.57 | 0.00 | | 20.59 | 7.27 |  |
| BM2 | 0.10 | 49.31 | 1.58 | 0.30 | | 18.25 | 8.59 |  |
| BM3 | 0.00 | 61.02 | 1.65 | 0.00 | | 22.31 | 18.09 |  |
| CBM1 | 0.00 | 0.95 | 1.35 | 0.00 | | 0.05 | 4.38 |  |
| CBM2 | 1.57 | 0.98 | 1.53 | 0.06 | | 0.10 | 7.46 |  |
| BBT1 | 0.00 | 0.00 | 1.58 | 0.42 | | 0.00 | 1.84 |  |
| BBT2 | 0.00 | 0.00 | 1.58 | 0.35 | | 0.00 | 1.78 |  |
| BT1 | 9.29 | 59.82 | 2.50 | 2.46 | | 29.30 | 10.48 |  |
| BT2 | 6.05 | 54.66 | 2.50 | 1.39 | | 23.25 | 12.15 |  |
| BT3 | 2.18 | 55.94 | 1.95 | 0.09 | | 27.58 | 32.66 |  |
| CBT1 | 0.00 | 0.74 | 1.91 | 0.00 | | 0.00 | 2.31 |  |
| CBT2 | 0.00 | 0.83 | 2.12 | 0.00 | | 0.00 | 2.45 |  |

**Table S2.** Total ammonifiable nitrogen and associated ammonium and ammonia concentrations in reactors at final time point

| Reactors: | TAN  (mg/L) | pH | f_NH_4_^+^ | f_NH_3_ | [NH_4_^+^]  (mg/L) | [NH_3_]  (mg/L) |
| --- | --- | --- | --- | --- | --- | --- |
| BAM1 | 1528.20 | 5.30 | 0.99989 | 0.00011 | 1528 | 0.18 |
| BAM2 | 1413.71 | 5.30 | 0.99989 | 0.00011 | 1414 | 0.16 |
| AM1 | 1714.04 | 5.30 | 0.99989 | 0.00011 | 1714 | 0.20 |
| AM2 | 1700.04 | 5.30 | 0.99989 | 0.00011 | 1700 | 0.20 |
| AM3 | 1749.52 | 5.30 | 0.99989 | 0.00011 | 1749 | 0.20 |
| CAM1 | 157.14 | 5.30 | 0.99989 | 0.00011 | 157 | 0.02 |
| CAM2 | 164.38 | 5.30 | 0.99989 | 0.00011 | 164 | 0.02 |
| BAT1 | 559.86 | 5.30 | 0.99989 | 0.00011 | 560 | 0.06 |
| BAT2 | 588.08 | 5.30 | 0.99989 | 0.00011 | 588 | 0.07 |
| AT1 | 1074.24 | 5.30 | 0.99989 | 0.00011 | 1074 | 0.12 |
| AT2 | 1142.35 | 5.30 | 0.99989 | 0.00011 | 1142 | 0.13 |
| AT3 | 1043.85 | 5.30 | 0.99989 | 0.00011 | 1044 | 0.12 |
| CAT1 | 385.57 | 5.30 | 0.99989 | 0.00011 | 386 | 0.04 |
| CAT2 | 359.62 | 5.30 | 0.99989 | 0.00011 | 360 | 0.04 |
| BBM1 | 2191.90 | 9.20 | 0.52301 | 0.47699 | 1146 | 1045.51 |
| BBM2 | 2019.39 | 9.20 | 0.52301 | 0.47699 | 1056 | 963.23 |
| BM1 | 2182.93 | 9.20 | 0.52301 | 0.47699 | 1141 | 1041.24 |
| BM2 | 2264.96 | 9.20 | 0.52301 | 0.47699 | 1185 | 1080.36 |
| BM3 | 2311.85 | 9.20 | 0.52301 | 0.47699 | 1209 | 1102.73 |
| CBM1 | 311.56 | 9.20 | 0.52301 | 0.47699 | 163 | 148.61 |
| CBM2 | 297.82 | 9.20 | 0.52301 | 0.47699 | 155 | 142.06 |
| BBT1 | 1466.83 | 9.20 | 0.52301 | 0.47699 | 767 | 699.66 |
| BBT2 | 1898.98 | 9.20 | 0.52301 | 0.47699 | 993 | 905.79 |
| BT1 | 2668.46 | 9.20 | 0.52301 | 0.47699 | 1396 | 1272.83 |
| BT2 | 2614.33 | 9.20 | 0.52301 | 0.47699 | 1367 | 1247.01 |
| BT3 | 2746.12 | 9.20 | 0.52301 | 0.47699 | 1436 | 1309.87 |
| CBT1 | 398.42 | 9.20 | 0.52301 | 0.47699 | 208 | 190.04 |
| CBT2 | 387.15 | 9.20 | 0.52301 | 0.47699 | 203 | 184.67 |

**Table S3.** Carbon balance details of reactors at initial and final time points

|  | Initial (TC as % initial TC) | | | | Final (TC as % initial TC) | | | | | Closure |
| --- | --- | --- | --- | --- | --- | --- | --- | --- | --- | --- |
| Reactors: | **Inoculum** | **Alkalinity** | **Duckweed** | **Total** | **Soluble** | **Particulate** | **Solid** | **Gaseous** | **Total** | **(%)** |
| BAM1 | 0 | 0 | 100 | 100 | 33.8 | 0 | 46.3 | 6.5 | 86.6 | 86.6 |
| BAM2 | 0 | 0 | 100 | 100 | 34.6 | 0 | 46.2 | 6.5 | 87.3 | 87.3 |
| AM1 | 11 | 0 | 89 | 100 | 14.3 | 15.4 | 48.9 | 13.7 | 92.4 | 92.4 |
| AM2 | 11 | 0 | 89 | 100 | 14.9 | 18.1 | 28.2 | 16 | 77.2 | 77.2 |
| AM3 | 11 | 0 | 89 | 100 | 15.2 | 8.1 | 41.6 | 14.3 | 79.2 | 79.2 |
| BAT1 | 0 | 0 | 100 | 100 | 30.3 | 0 | 49.5 | 6.4 | 86.3 | 86.3 |
| BAT2 | 0 | 0 | 100 | 100 | 27.1 | 0 | 59.4 | 4.5 | 91 | 91 |
| AT1 | 11 | 0 | 89 | 100 | 19.9 | 3 | 65.9 | 5.4 | 94.3 | 94.3 |
| AT2 | 11 | 0 | 89 | 100 | 22.9 | 5.5 | 56.4 | 5.8 | 90.6 | 90.6 |
| AT3 | 11 | 0 | 89 | 100 | 20.6 | 5.3 | 58.9 | 5.9 | 90.7 | 90.7 |
| BBM1 | 0 | 5 | 95 | 100 | 43.3 | 0 | 51.3 | 0.1 | 94.7 | 94.7 |
| BBM2 | 0 | 5 | 95 | 100 | 52.8 | 1.4 | 43.3 | 0.1 | 97.5 | 97.5 |
| BM1 | 10.2 | 4.5 | 85.2 | 100 | 51.4 | 0 | 26.9 | 2.3 | 80.6 | 80.6 |
| BM2 | 10.2 | 4.5 | 85.2 | 100 | 52.9 | 0 | 27.9 | 2.4 | 83.2 | 83.2 |
| BM3 | 10.2 | 4.5 | 85.2 | 100 | 53.9 | 6.9 | 27.5 | 3.6 | 91.9 | 91.9 |
| BBT1 | 0 | 5 | 95 | 100 | 42.4 | 19 | 42.6 | 0.1 | 104 | 104 |
| BBT2 | 0 | 5 | 95 | 100 | 41.2 | 18.2 | 40.9 | 0.1 | 100.3 | 100.3 |
| BT1 | 10.2 | 4.5 | 85.2 | 100 | 53.1 | 5.5 | 36.1 | 3.2 | 98 | 98 |
| BT2 | 10.2 | 4.5 | 85.2 | 100 | 50 | 5.2 | 41.3 | 2.8 | 99.3 | 99.3 |
| BT3 | 10.2 | 4.5 | 85.2 | 100 | 48.7 | 6.9 | 35.4 | 4.3 | 95.3 | 95.3 |

| REACTORS: | Daily Pressures (psi) | | | | | | | | | | | | | | |
| --- | --- | --- | --- | --- | --- | --- | --- | --- | --- | --- | --- | --- | --- | --- | --- |
|  | **0.5** | **1** | **2** | **3** | **4** | **5** | **6** | **7** | **9** | **11** | **13** | **15** | **17** | **19** | **21** |
| BAM1 | 4.17 | 0 | 0.77 | 1.62 | 0 | 2.13 | 2.58 | 2.47 | 3.67 | 2.58 | 1.16 | 0.84 | 0.07 | -0.28 | 0.77 |
| BAM2 | 4.13 | 0 | 1.36 | 1.9 | 0 | 2.08 | 2.83 | 2.68 | 3.31 | 2.25 | 1.69 | 1.35 | 0.17 | 0 | 0.65 |
| AM1 | 5.22 | 1.27 | 1.58 | 2.22 | 1.79 | 0.9 | 1.62 | 1.24 | 1.41 | 2.33 | 5.38 | 5.65 | 12.75 | 7.13 | 6.25 |
| AM2 | 5.45 | 1.34 | 2.57 | 2.65 | 1.74 | 1.98 | 1.88 | 1.48 | 2.4 | 5.12 | 9.67 | 12.25 | 7.98 | 4.22 | 4 |
| AM3 | 4.9 | 1.27 | 1.72 | 3.36 | 1.74 | 1.82 | 1.54 | 1.07 | 1.55 | 2.93 | 6.16 | 12.22 | 9.75 | 0.3 | 5.95 |
| CAM1 | -0.5 | 0.91 | -0.09 | 0.09 | -0.21 | 0.22 | 0.03 | 0.19 | -0.34 | 0 | 0.07 | -0.25 | -0.59 | -0.63 | -0.23 |
| CAM2 | 0 | 0.72 | -0.08 | 0.12 | -0.28 | 0.15 | 0.14 | 0.14 | -0.18 | -0.06 | 0.07 | 0.09 | -0.29 | -0.32 | 0 |
| BAT1 | 2.86 | 2.4 | 2.75 | 4.22 | 4.64 | 2.85 | 1.94 | 2.25 | 9.15 | 6.16 | 3.72 | 5.23 | 3.02 | 3.93 | 1.66 |
| BAT2 | 4.54 | 2.4 | 2.39 | 1.98 | 1.37 | 0.73 | 1.44 | 1.5 | 4.28 | 2.58 | 2.26 | 3.8 | 3.48 | 4.13 | 2.05 |
| AT1 | 14.9 | 1.84 | 8.67 | 7.04 | 2.51 | 2.4 | 1.18 | 1 | 0.16 | 0.14 | 0 | 1.01 | 0.92 | 3.4 | 0.11 |
| AT2 | 14.3 | 2.92 | 9.43 | 8.26 | 2.37 | 1.69 | 1.27 | 0.77 | -0.22 | -0.26 | -0.43 | -0.13 | -0.05 | 2.19 | 0 |
| AT3 | 14.5 | 3.33 | 7.22 | 7.19 | 2.69 | 1.87 | 0.98 | 1.12 | 1.63 | 1.15 | 0.87 | 2.76 | 0.71 | 0.84 | -0.31 |
| CAT1 | 1.89 | 0.2 | 0.89 | 0.79 | -0.59 | 0.62 | -0.07 | -0.59 | -0.18 | -0.32 | -0.78 | -0.31 | -0.24 | -0.19 | -0.38 |
| CAT2 | 1.01 | 0.2 | 0.8 | 0.2 | -0.63 | 0.51 | -0.29 | -0.21 | -0.08 | -0.29 | -0.98 | -0.31 | -0.31 | 0 | -0.2 |
| BBM1 | N/A | 1.05 | 0.82 | -0.24 | -0.06 | -0.63 | 0.14 | 0.06 | -0.06 | 0.53 | -0.05 | -0.14 | -0.11 | 0.45 | 0.11 |
| BBM2 | N/A | 1.01 | 1.19 | 0.34 | -0.06 | -1.1 | -0.56 | -0.71 | -0.49 | 0.16 | -0.05 | 0.07 | 0.22 | 0.14 | 0.14 |
| BM1 | N/A | 1.21 | 2 | 2.16 | -0.06 | 0.63 | 1.23 | 0.84 | 1.06 | 1.32 | 0.27 | 0.84 | 0.21 | 0.49 | 0.4 |
| BM2 | N/A | 1.23 | 2.19 | 1.87 | -0.64 | -0.27 | 0 | 1.11 | 0.98 | 0.62 | 0.45 | 0.71 | 0.56 | 0.55 | 0.63 |
| BM3 | N/A | 1.18 | 1.98 | 2.03 | 4.41 | 4.82 | -0.6 | -0.32 | -0.32 | 1.07 | 0.24 | 0.48 | 0.31 | 0.48 | 0.61 |
| CBM1 | N/A | 0.77 | 0.44 | -0.29 | 0 | -0.45 | -0.06 | -0.14 | -0.14 | 0.2 | -0.27 | -0.27 | -0.11 | -0.05 | 0.06 |
| CBM2 | N/A | 0.73 | 0.05 | 0.06 | 0.27 | -0.3 | 0 | 0 | -0.05 | 0.34 | -0.29 | -0.26 | -0.14 | -0.09 | 0.09 |
| BBT1 | N/A | 1.85 | 0.05 | 0.34 | 0.3 | 0.4 | -0.27 | 0.07 | 0.16 | 0.09 | 0.16 | -0.77 | 0.28 | 0.86 | 0.89 |
| BBT2 | N/A | 1.74 | 0.5 | 0.36 | 0.18 | 0.34 | 0.16 | -0.13 | -0.35 | 0 | 0.24 | -0.2 | 0.59 | 0.58 | 1.27 |
| BT1 | N/A | 2.41 | 1.9 | 2.4 | 0.25 | 0.63 | 0.92 | 0.77 | 0.8 | 0.83 | 0.97 | 0.5 | 0.62 | 0.03 | 0.03 |
| BT2 | N/A | 2.08 | 1.76 | 2.54 | -1.08 | -0.71 | -1.01 | -0.35 | 0.59 | 0.30 | 0.19 | 0.76 | 0.09 | 0.24 | 0.43 |
| BT3 | N/A | 2.39 | 2.41 | 7.49 | 4.74 | 6.13 | -2.81 | -2.37 | -2.37 | 0.56 | 0.82 | -0.47 | 0.39 | 0.39 | 0.02 |
| CBT1 | N/A | 1.48 | 0.62 | 0.66 | 0.41 | 0 | 0 | 0.09 | -0.05 | 0.24 | 0.08 | -0.76 | -0.2 | -0.26 | 0 |
| CBT2 | N/A | 1.21 | 0.71 | 0.19 | 0.25 | 0 | -0.12 | 0 | -0.56 | -0.37 | -0.57 | -1.35 | -0.58 | -0.84 | -0.42 |

**Table S4.** Headspace pressure in reactors over time.

**Box S1.** One-way ANOVA and TUKEY comparison results of VFA Yields achieved in active (with inoculum) reactors

| One-way ANOVA: AM, AT, BM, BT  Method   \| Null hypothesis \| All means are equal \| \| --- \| --- \| \| Alternative hypothesis \| Not all means are equal \| \| Significance level \| α = 0.05 \|   *Equal variances were assumed for the analysis.*  Factor Information   \| Factor \| Levels \| Values \| \| --- \| --- \| --- \| \| Factor \| 4 \| AM, AT, BM, BT \|   Analysis of Variance   \| Source \| DF \| Seq SS \| Contribution \| Adj SS \| Adj MS \| F-Value \| P-Value \| \| --- \| --- \| --- \| --- \| --- \| --- \| --- \| --- \| \| Factor \| 3 \| 0.177185 \| 98.93% \| 0.177185 \| 0.059062 \| 247.40 \| 0.000 \| \| Error \| 8 \| 0.001910 \| 1.07% \| 0.001910 \| 0.000239 \|  \|  \| \| Total \| 11 \| 0.179095 \| 100.00% \|  \|  \|  \|  \|   Model Summary   \| S \| R-sq \| R-sq(adj) \| PRESS \| R-sq(pred) \| \| --- \| --- \| --- \| --- \| --- \| \| 0.0154510 \| 98.93% \| 98.53% \| 0.0042972 \| 97.60% \|   Means   \| Factor \| N \| Mean \| StDev \| 95% CI \| \| --- \| --- \| --- \| --- \| --- \| \| AM \| 3 \| 0.04713 \| 0.00388 \| (0.02656, 0.06770) \| \| AT \| 3 \| 0.09989 \| 0.00832 \| (0.07932, 0.12047) \| \| BM \| 3 \| 0.3322 \| 0.0294 \| (0.3116, 0.3528) \| \| BT \| 3 \| 0.29169 \| 0.00290 \| (0.27112, 0.31226) \|   *Pooled StDev = 0.0154510*  Tukey Pairwise Comparisons  Grouping Information Using the Tukey Method and 95% Confidence   \| Factor \| N \| Mean \| Grouping \| \| \| \| \| --- \| --- \| --- \| --- \| --- \| --- \| --- \| \| BM \| 3 \| 0.3322 \| A \|  \|  \|  \| \| BT \| 3 \| 0.29169 \|  \| B \|  \|  \| \| AT \| 3 \| 0.09989 \|  \|  \| C \|  \| \| AM \| 3 \| 0.04713 \|  \|  \|  \| D \|   *Means that do not share a letter are significantly different.* |
| --- | --- | --- | --- | --- | --- | --- | --- | --- | --- | --- | --- | --- | --- | --- | --- | --- | --- | --- | --- | --- | --- | --- | --- | --- | --- | --- | --- | --- | --- | --- | --- | --- | --- | --- | --- | --- | --- | --- | --- | --- | --- | --- | --- | --- | --- | --- | --- | --- | --- | --- | --- | --- | --- | --- | --- | --- | --- | --- | --- | --- | --- | --- | --- | --- | --- | --- | --- | --- | --- | --- | --- | --- | --- | --- | --- | --- | --- | --- | --- | --- | --- | --- | --- | --- | --- | --- | --- | --- | --- | --- | --- | --- | --- | --- | --- | --- | --- | --- | --- | --- | --- | --- | --- | --- | --- | --- | --- | --- | --- | --- | --- | --- | --- | --- |

**Box S2.** One-way ANOVA and TUKEY comparison results of VFA yields achieved in blank (without inoculum) reactors

| One-way ANOVA: BAM, BAT, BBM, BBT  Method   \| Null hypothesis \| All means are equal \| \| --- \| --- \| \| Alternative hypothesis \| Not all means are equal \| \| Significance level \| α = 0.05 \| \| Rows unused \| 4 \|   *Equal variances were assumed for the analysis.*  Factor Information   \| Factor \| Levels \| Values \| \| --- \| --- \| --- \| \| Factor \| 4 \| BAM, BAT, BBM, BBT \|   Analysis of Variance   \| Source \| DF \| Seq SS \| Contribution \| Adj SS \| Adj MS \| F-Value \| P-Value \| \| --- \| --- \| --- \| --- \| --- \| --- \| --- \| --- \| \| Factor \| 3 \| 0.028830 \| 87.80% \| 0.028830 \| 0.009610 \| 9.59 \| 0.027 \| \| Error \| 4 \| 0.004007 \| 12.20% \| 0.004007 \| 0.001002 \|  \|  \| \| Total \| 7 \| 0.032837 \| 100.00% \|  \|  \|  \|  \|   Model Summary   \| S \| R-sq \| R-sq(adj) \| PRESS \| R-sq(pred) \| \| --- \| --- \| --- \| --- \| --- \| \| 0.0316508 \| 87.80% \| 78.64% \| 0.0160283 \| 51.19% \|   Means   \| Factor \| N \| Mean \| StDev \| 95% CI \| \| --- \| --- \| --- \| --- \| --- \| \| BAM \| 2 \| 0.18705 \| 0.00930 \| (0.12492, 0.24919) \| \| BAT \| 2 \| 0.0986 \| 0.0338 \| (0.0365, 0.1607) \| \| BBM \| 2 \| 0.2187 \| 0.0450 \| (0.1566, 0.2809) \| \| BBT \| 2 \| 0.0739 \| 0.0275 \| (0.0117, 0.1360) \|   *Pooled StDev = 0.0316508*  Tukey Pairwise Comparisons  Grouping Information Using the Tukey Method and 95% Confidence   \| Factor \| N \| Mean \| Grouping \| \| \| --- \| --- \| --- \| --- \| --- \| \| BBM \| 2 \| 0.2187 \| A \|  \| \| BAM \| 2 \| 0.18705 \| A \| B \| \| BAT \| 2 \| 0.0986 \| A \| B \| \| BBT \| 2 \| 0.0739 \|  \| B \|   *Means that do not share a letter are significantly different.* |
| --- | --- | --- | --- | --- | --- | --- | --- | --- | --- | --- | --- | --- | --- | --- | --- | --- | --- | --- | --- | --- | --- | --- | --- | --- | --- | --- | --- | --- | --- | --- | --- | --- | --- | --- | --- | --- | --- | --- | --- | --- | --- | --- | --- | --- | --- | --- | --- | --- | --- | --- | --- | --- | --- | --- | --- | --- | --- | --- | --- | --- | --- | --- | --- | --- | --- | --- | --- | --- | --- | --- | --- | --- | --- | --- | --- | --- | --- | --- | --- | --- | --- | --- | --- | --- | --- | --- | --- | --- | --- | --- | --- | --- | --- | --- | --- | --- | --- | --- | --- | --- | --- | --- | --- | --- | --- | --- |
